# Advancements in deep learning for early diagnosis of Alzheimer’s disease using multimodal neuroimaging: challenges and future directions

**DOI:** 10.3389/fninf.2025.1557177

**Published:** 2025-05-02

**Authors:** Muhammad Liaquat Raza, Syed Tawassul Hassan, Subia Jamil, Noorulain Hyder, Kinza Batool, Sajidah Walji, Muhammad Khizar Abbas

**Affiliations:** ^1^Department of Infection Prevention Control, Ministry of National Guard Health Affairs (MNGHA), Riyadh, Saudi Arabia; ^2^IPC, King Abdullah International Medical Research Center (KAIMRC), Riyadh, Saudi Arabia; ^3^IPC, King Saud bin Abdulaziz University for Health Sciences (KSAU-HS), Riyadh, Saudi Arabia; ^4^Medicine, Liaquat University of Medical and Health Sciences (LUMHS), Jamshoro, Pakistan; ^5^Pharmacology, Faculty of Pharmacy, Jinnah University for Women (JUW), Karachi, Pakistan; ^6^Department of Pharmacology, Faculty of Pharmacy, Hamdard University (FoP-HU), Karachi, Pakistan; ^7^Karachi Medical and Dental College (KMDC), Karachi, Pakistan; ^8^PNEC, National University of Sciences and Technology (NUST), Karachi, Pakistan

**Keywords:** Alzheimer’s disease, deep learning, multimodal neuroimaging, Disease progression prediction, brain imaging analysis

## Abstract

**Introduction:**

Alzheimer’s disease is a progressive neurodegenerative disorder challenging early diagnosis and treatment. Recent advancements in deep learning algorithms applied to multimodal brain imaging offer promising solutions for improving diagnostic accuracy and predicting disease progression.

**Method:**

This narrative review synthesizes current literature on deep learning applications in Alzheimer’s disease diagnosis using multimodal neuroimaging. The review process involved a comprehensive search of relevant databases (PubMed, Embase, Google Scholar and ClinicalTrials.gov), selection of pertinent studies, and critical analysis of findings. We employed a best-evidence approach, prioritizing high-quality studies and identifying consistent patterns across the literature.

**Results:**

Deep learning architectures, including convolutional neural networks, recurrent neural networks, and transformer-based models, have shown remarkable potential in analyzing multimodal neuroimaging data. These models can effectively process structural and functional imaging modalities, extracting relevant features and patterns associated with Alzheimer’s pathology. Integration of multiple imaging modalities has demonstrated improved diagnostic accuracy compared to single-modality approaches. Deep learning models have also shown promise in predictive modeling, identifying potential biomarkers and forecasting disease progression.

**Discussion:**

While deep learning approaches show great potential, several challenges remain. Data heterogeneity, small sample sizes, and limited generalizability across diverse populations are significant hurdles. The clinical translation of these models requires careful consideration of interpretability, transparency, and ethical implications. The future of AI in neurodiagnostics for Alzheimer’s disease looks promising, with potential applications in personalized treatment strategies.

## Graphical abstract

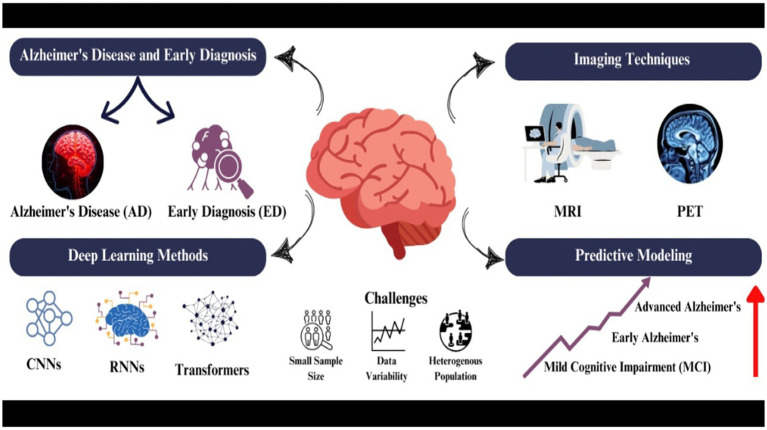

## Highlights

- Integration of MRI, PET, and fMRI with deep learning models for early AD detection.
- Exploration of CNNs, RNNs, and transformers in analyzing brain imaging data.
- Use of AI to predict disease progression, differentiate MCI and AD stages, and identify biomarkers.
- Addressing issues like data heterogeneity, small sample sizes, and generalizability.
- Discussing model interpretability, regulatory considerations, and AI’s role in personalized AD treatments.

## 1 Introduction

### 1.1 Overview of Alzheimer’s disease and the importance of early diagnosis

Alzheimer’s Disease (AD) is defined as a neurodegenerative disorder characterized by cognitive decline, memory loss, and behavioural changes which cannot be reversed (Porsteinsson et al., 2021). There is no cure for this disease but treatments are available which can decline its progression (Raza et al., 2024a; Atri, 2019). It is one of the most common causes of dementia, affecting 50% of the 85-year-old age group population (Aramadaka et al., 2023). AD causes the degeneration of neurons (Raza et al., 2024b), specifically those that are involved in memory and intellectual functions (Atri, 2019).

It’s very important to diagnose AD before its onset. While there is no cure for AD, early diagnosis allows health professionals to take timely intervention (Raza et al., 2024a) and screening to manage the disease so that its progressive nature can be slowed down (Porsteinsson et al., 2021). It can also help patients as well as care providers in a way that they can plan for the necessary lifestyle changes in future to make the patient’s life quality better (Porsteinsson et al., 2021). According to researchers, rolling back the diagnosis by 5 years could reduce the frequency of AD by 50% (Amira et al., 2015). Challenges have often come along with the early diagnosis, which includes limitation of time for clinicians, proper diagnosis of AD pathology and misdiagnosis of the symptoms, which are similar to the normal ageing process (Porsteinsson et al., 2021).

Despite facing such challenges, early diagnosis and continuous research led to the development of advanced diagnostic tools and biological markers. Many diagnostic tests have been investigated, such as neuropsychological and neuroimaging tests, and their working with machine learning (ML) and deep learning (DL) showed promising results in diagnosing AD at its early stages (Ding et al., 2019; Feng et al., 2020). The use of Electroencephalography (EEG) together with ML has shown high accuracy in diagnosing AD patients (Pirrone et al., 2022). Neuropsychological testing focuses on dual-task performance and active inhibition to catch AD in its early stages while differentiating it from the control group (Toepper et al., 2008).

Magnetic Resonance Imaging (MRI) and Computer-Aided Diagnostic (CAD) systems, which are classified under Neuroimaging techniques, were found to achieve good accuracy in the early diagnosis of AD. MRI and CAD use Deep Neural Networks (DNN) and Convolutional Neural Networks (CNNs), which are advanced subfields of ML; by using these approaches, MRI and CAD detect AD-related patterns with accuracy higher than 90% (Shah et al., 2024; Amira et al., 2015; Rabeh et al., 2023).

### 1.2 Role of neuroimaging in Alzheimer’s disease diagnosis

Neuroimaging is defined as the process of image production of the structure or the activity of the central nervous system, primarily the brain, through techniques like MRI or computerized tomography (CT). However, neuroimaging plays an important role in diagnosing AD. Positron emission tomography (PET) and single-photon emission computed tomography (SPECT) are the techniques which have been useful in detecting structural and functional changes in the brain that are associated with AD. These techniques have greatly contributed to AD research and diagnosis (Weinstein et al., 1993).

MRI can detect fine structural and functional changes in different regions of the brain, including the integrity of the blood–brain barrier, through dynamic contrast-enhanced MRI (DCE-MRI) technique (Raja et al., 2018). MRI has been widely used in AD diagnosis. Brain atrophy patterns, which are the characteristics of the AD process, can be detected through volumetric MRI (Zhou et al., 2024). Neuroimaging data has a multi-dimensional nature; this can present analytical issues (Shu, 2016). To address this, researchers have developed voxel-level analyses, region of interest (ROI) studies and more advanced procedures like seed-voxel and multivariate techniques (Albajes-Eizagirre et al., 2019; Habeck et al., 2010). These techniques can assist in extracting relevant features and patterns from brain images.

The emergence of AI and advances in ML have transformed the analysis of neuroimaging in diagnosing AD. These advanced computational tools give more precise diagnoses of AD patients and disease progression. Deep learning method CNNs extract relevant features from brain images while classifying them into different stages of cognitive impairment (Parmar et al., 2020). However, this association of AI and ML provides diagnostic accuracy and efficient analyses of radiographic data and contributes to the development of precision medicine approaches for AD (Mirkin and Albensi, 2023; Jo et al., 2019).

Longitudinal data analysis of neuroimaging data is another important aspect of AD discussion. It allows for the study of disease progression over time (Aberathne et al., 2023). It can also help in identifying vulnerable brain regions and determining threshold values for biomarkers such as plaques, tangles, and neurodegeneration (Aberathne et al., 2023). The diverse effects of AD on the brain have been studied by employing normative modelling techniques. These techniques analyze specific individual changes across multiple imaging biomarkers that reveal the effect of AD on the brain (Verdi et al., 2024). While neuroimaging biomarkers have shown promise in AD diagnosis, they are often limited in clinical application due to their high cost and, in some cases, invasive nature (Khan and Alkon, 2015). Research is needed for more accessible and cost-effective biomarkers, such as blood-based markers, which can introduce neuroimaging techniques in low and middle-income healthcare settings.

Neuroimaging when associated with AI and ML can be a great tool for the early and more accurate diagnosis of AD as It provides valuable insights into brain structure and function that helps in monitoring disease progression. Research is needed to deal with the challenges in standardizing methods and validating findings across different populations.

### 1.3 Advancements in deep learning for brain imaging analysis

Deep learning (DL) is a type of machine learning (ML) that utilize artificial neural networks having multiple layers of processing and extracting high level features from the data while performing complex task like classification, regression and representation of the data. DL has made great advancements in brain imaging analysis serving the field of neuroscience and medical imaging.

Artificial neural networks such as CNNs, which are the type of DL algorithm used to identify patterns in images and perform computer vision tasks, showed success in diagnosing and classifying the imaging data for the early detection and classification of AD (Wen et al., 2020). CNNs can be applied to MRI and PET images for the identification and classification of different structures enhancing the accuracy of diagnosis of AD (Wen et al., 2020). It provides great help for the radiologist to interpret complex cases more effectively and decrease their workload through its automation process. However, the circle of DL is not limited to just AD. It extends far beyond it. It works with several other brain disorders, such as Parkinson’s disease, autism spectrum disorder, and Schizophrenia (Zhang et al., 2020a).

DL methods have bypassed the traditional ML techniques in diagnosis through computer-aided techniques, and generative adversarial networks (GANs) have been explored to overcome the limitations associated with data availability in brain imaging studies (Galić et al., 2023; Logan et al., 2021). DL methods, when compared to standard ML methods, were found to have a better-scaling potential and their ability to make the most of nonlinearities in neuroimaging data to make more effective representations of tasks for better characterization of the human brain (Abrol et al., 2021). From adult to pediatric medical imaging, DL has been instrumental in detecting neurological developmental disorders by identifying brain and tissue structures while predicting the outcomes (Hu et al., 2023).

However, DL approaches have greatly covered several aspects that include brain imaging, early detection of disorder, classification and data representation but further research is needed to address the interpretation issues and medical images variability to utilize the full potential of DL.

### 1.4 Objectives of the review

- To provide an overview of AD and emphasize the significance of early diagnosis in improving patient outcomes.
- To explore the role of neuroimaging techniques, such as MRI and PET, in the diagnosis and monitoring of AD.
- To review advancements in deep learning methodologies applied to brain imaging analysis, particularly for the early detection and progression of AD.
- To discuss various deep learning architectures, including CNNs, RNNs, transformers, and hybrid models, and their applications in structural and functional neuroimaging for AD diagnosis.
- To evaluate the role of MRI and PET fusion and other structural imaging and functional imaging techniques in providing early diagnosis of AD.
- To analyze the use of deep learning methods in predictive modelling to track disease progression, find biomarkers and distinguish MCI from AD.
- To underline the issues in neuroimaging data standardization, including variability in imaging protocols, limited data availability, and generalizability across diverse populations.
- To provide suggestions for future research, such as the development of explainable deep learning models, multimodal data integration, and solutions for overcoming data-related limitations.

## 2 Convolutional neural network for structural imaging analysis

CNNs are the cornerstone of deep learning models capable of handling high-dimensional data such as neuroimages. Its basic framework consists of an input layer, some middle layers, and an output layer. The hidden layers include the convolutional layers (C-layers), the subsampling layers (S-layers), and the fully connected layers (FC-layers). All these layers cooperate to identify the valuable insight in the data, to decrease the data volume, and to categorize the data.

As the CNNs advance, they become refined and incorporate new changes in the imaging equipment, such as MRI and PET scans. However, when trained on larger data sets, it is boosted to a great extent. The fact that CNNs can learn directly from the images means that the features can be learned automatically without having to handcraft them. This potential makes them suitable for various applications, making them effective in measuring the accurate staging of Alzheimer’s disease.

In AD studies, CNNs are trained to recognize morphological and metabolic alterations of the brain by concentrating on specific areas, including the hippocampus, amygdala, and insula. Their capability to learn from these areas of the damaged brain makes them crucial for early-stage detection and diagnosis. For instance, CNNs have the potential to recognize hippocampal and cortical atrophy from MRI or abnormalities in glucose metabolism and amyloid deposition from PET. Due to the ability of CNNs to handle massive high-dimensional data sets such as the ones from ADNI and OASIS, which contain neuroimaging and clinical information, CNNs are employed.

CNNs can enhance the accuracy of detecting Alzheimer’s even before the appearance of clinical symptoms by learning from a lot of data. They also help in the creation of predictive models as well as individualized interventional approaches. These deep-learning models are more convenient for medical images since they can learn hierarchical features (Saleem et al., 2022). Some of the CNN models that have been employed in the diagnosis of AD include AlexNet and DenseNet, among others. These models not only distinguish between a healthy person and a patient but can also assess the patient’s condition as in either the early stage of the disease or the advanced stage, incorporating the mild cognitive impairment (MCI) and the early stage of AD. For instance, AlexNet has exhibited remarkable results in analyzing MRI images and thus set a high standard in this field (Dakdareh and Abbasian, 2024).

### 2.1 Recurrent neural networks (RNNs) for longitudinal data and temporal analysis

Recurrent Neural Networks (RNNs) could be considered one of the best approaches to processing longitudinal and temporal physiological data because of their capability to manage sequential inputs and temporal dependencies. DNNs, CNNs, or other machine learning models could be used in analyzing dynamic systems, but RNNs are best suited for this purpose. The features they consider are both the current and previous sequences of features, which is useful when faced with input sequences of varying lengths (Mao and Sejdic, 2023).

In the case of MCI to AD conversion, annual NP test data are used by RNNs to incorporate temporal patterns of the disease. This allows RNN models to capture the change from MCI to AD over several years and indicates the patient’s future prognosis. It can be concluded that the proposed RNN-based models are more effective than traditional models (Table 1), including logistic regression, in the case of using fewer features and without using expensive data such as MRI images. This can make a difference in the quality of the patient and the cost of the treatment; in fact, it has the potential to place RNN-based predictions as the new generation of Alzheimer’s treatment.

**Table 1:** Comparative analysis of deep learning and traditional models.

Approach	Examples	Accuracy (%)	AUC	Key Features	Reference
Traditional ML	SVM, Random Forest, Decision Trees	94.5%	0.96	Requires manual feature extraction; interpretable; data-efficient	Alshamlan et al. (2024)
Deep Learning (CNNs, RNNs, Transformers)	AlexNet, DenseNet, Vision Transformer, 3D-CNN	94–99	0.91–0.99	End-to-end learning; automatic feature extraction; scalable for large datasets	Alsubaie et al. (2024); Khan and Saddam (2024)
Hybrid DL Models	CNN + RNN, Transformer-CNN Fusion	91–99	0.937	Multimodal learning; enhanced accuracy by combining imaging and clinical data	Asaduzzaman et al. (2025); Vashishtha et al. (2023)

Contrary to other methods that depend on costly and detailed imaging studies, RNNs use easily accessible NP tests to make relevant and timely predictions based on patients’ records. In this manner, RNNs can predict the stages of MCI to AD over the next few years, which will also be more inexpensive and less invasive than the current diagnosis and management of the disease (Park et al., 2024) In their present form, RNNs could be more effective than traditional models like logistic regression, even with few input data. This advancement can also improve the lives of the patients and the healthcare system, which results in a decline in costs for the treatment of AD (Al Olaimat and Bozdag, 2024).

### 2.2 Transformer and attention mechanisms for multimodal data integration

Transformers have played a crucial role in the development of AI, especially in dealing with sequential data using innovative approaches to the self-attention mechanism. This mechanism allows the transformers to work on the parts of a sequence that are relevant to the task at hand, irrespective of their location Table 2. Therefore, the models are capable of learning long-range dependencies (Madan et al., 2024). The transformers were initially designed for natural language processing and have been adopted in healthcare, especially in such areas as the diagnosis. AD is complex and therefore has multiple etiologies, and the progression of this disease needs several data sets for better prediction.

**Table 2:** Quantitative performance metrics for deep learning models in Alzheimer’s diagnosis.

Model	Modality	Accuracy (%)	Sensitivity (%)	Specificity (%)	AUC	Reference
CNN (AlexNet, DenseNet, ResNet)	MRI	89.4–98.9	86.7–98.9	84–98.8	—	Feng et al. (2020)
3D ResAttNet	MRI	84–93.44	—	—	81.8–96.7	Wang Y. et al. (2024)
RNN (LSTM, GRU-based)	Longitudinal Data	75.57	—	—	—	Airlangga (2024)
Transformer (Vision Transformer, Med-PLM, BRLTM)	Multimodal Data	92.14%	93.27%	89.95%	—	Zhao et al. (2024)
Hybrid Models (CNN + RNN, HiLCAE +3D-VGG16, BGRU + DenseNet)	MRI + PET	98.8%	100%	76%	—	Khatun et al. (2023)

In the framework of Alzheimer’s diagnosis, transformers exhibit tremendous potential in aggregating various types of data such as EHRs, genetics, and imaging. Transformers are especially effective in dealing with multimodal data since Alzheimer’s presents differently in every patient. This includes data, in the form of brain scans and genetic markers, which offer a more complete and specific view of the patient. For instance, some models like Med-PLM and BRLTM have incorporated diagnosis codes, medical histories, and even unstructured clinical notes to enhance the diagnostic abilities of the model, which shows the future of transformers in this domain.

Additionally, transformers are getting more recognition in medical imaging, especially Vision Transformers (ViTs), as they have the potential to handle larger effective receptive fields. This makes them capable of capturing more contextual information in medical images than the conventional CNNs; this is very important as it determines the overall diagnosis of the tissue and organs. Although they face difficulties in terms of computational intensity and the requirement of big data sets, transformers are being considered a great perspective in medical imaging as a potential way to improve the quality of healthcare, especially in the context of Alzheimer’s diagnosis (Tang et al., 2023).

### 2.3 Hybrid deep learning model combining multiple techniques

The hybrid deep learning models have enhanced the performance of diagnosing AD to the state where it has not been before through a combination of different architectures that increase the accuracy and reliability of the model. These models incorporate various models such as CNNs, Auto-Encoders (AEs), and RNNs to process multi-modal data, including MRI and PET images as well as speech samples (Saleem et al., 2022).

For instance, the 3D Explainable Residual Self-Attention Convolutional Neural Network (3D ResAttNet) uses a self-attention mechanism along with residual connections to improve attention towards important features while at the same time improving the training efficiency. Such models as sparse autoencoders (AEs) and capsule networks are applied for the refinement of feature extraction with the purpose of enhancement of detection. Further, the high-level layer concatenation autoencoder (HiLCAE) along with 3D-VGG16 has been used to improve diagnostic performance by using both MR and PET images. To examine the feasibility of AD, CNNs and RNNs are used to analyze speech information (Saleem et al., 2022).

Further, Bidirectional Gated Recurrent Units (BGRUs) combined with Dense Convolutional Networks (DenseNets) enable us to learn spatial and temporal patterns from the brain data. Other complicated structures like SBi-RNNs combined with 3D-CNNs, as well as Dense Connection CNNs with attention, have, however, been indicated to perform better than the traditional methods. These models can therefore be used to improve and expedite the diagnosis of AD, making the models relevant in any clinical setting. Nationally, models such as Bidirectional Gated Recurrent Units (BGRU) along with Dense Convolutional Networks (DenseNets) are capable of learning both spatial and temporal features from brain data. Other complex architectures, including Stacked Bidirectional Recurrent Neural Networks (SBi-RNNs) incorporated with 3D-CNNs and Densely Connected Convolutional Neural Networks (DCCNNs) with attention mechanisms, have been shown to outperform the conventional approaches (Saleem et al., 2022). These models provide a platform for enhanced and timely AD diagnosis, thus making them applicable in any given clinical setting.

One such model is the 3D Hybrid Compact Convolutional Transformer (3D HCCT), which is an advanced model for the AD classification from 3D MRI scans. These networks are convenient in learning the local features, while these are optimal when our focus is on the global forms and the long-term relations. The entity acquires the local features while ViTs are effective at capturing the global patterns and long-range dependencies. This architecture combines the two models to produce richer feature representations that are more discriminative and therefore achieve better accuracy, and sensitivity, in contrast to the traditional CNN-based models, specifically when tested on the Alzheimer’s Disease Neuroimaging Initiative (ADNI) data sets (Majee et al., 2024).

Also, the 3D HCCT’s ability to perform well on various data sets and its efficient architecture that reduces the consumption of resources makes it suitable for use in the clinical setting. Its ability to aid in the diagnosis of AD at an early stage and with a high level of accuracy shows how AI in healthcare can transform the healthcare industry and improve patient care.

## 3 Integration of structural and functional imaging

The fusion of resting-state functional MRI (rs-MRI) with Structural MRI has shown better outcomes in the early detection as well as staging of AD (Hojjati et al., 2018) the multimodal approaches demonstrates great promise in accuracy as compared to single modality approaches. For instance, combined sMRI and rs-fMRI modality outperformed sMRI (89%) and rs-fMRI (93%) alone and demonstrated 97% accuracy in diagnosing MCI converters against non-converters (Hojjati et al., 2018).

Likewise, combining MRI, fMRI, and cognitive test scores produced 95.65% accuracy in distinguishing AD patients from healthy controls (Dachena et al., 2020). Though, the advantages of combining functional and structural imaging can vary across patient categories. For example, integration improves differentiation between AD and behavioral variant frontotemporal dementia (bvFTD) but does not always significantly outperform structural measures alone when distinguishing AD from healthy controls (Bouts et al., 2018) Therefore, multimodal imaging approaches effectively capture complementary information about brain structure and function, enhancing diagnostic accuracy and strength. Additional research is required to optimize feature selection, integration methods, as well as clarification of the role of each modality across different patient populations.

### 3.1 MRI and PET fusion for early detection of Alzheimer’s disease

The combination of MRI and PET methods demonstrates better outcomes in the early detection of AD by synchronizing the structural revelations of MRI to the metabolic status captured by PET. This integration results in a more comprehensive view of brain changes. Studies have revealed that multimodal fusion approaches outperform individual modality methods in diagnostic accuracy (Castellano et al., 2024; Du et al., 2024a; Shukla et al., 2023). Noteworthy methods embrace the integration of gray matter (GM) areas from MRI with FDG-PET (Fluorodeoxyglucose-positron emission tomography) to create a “GM-PET” modality and a wavelet transform-based fusion process combined with deep learning for categorization. These techniques have attained up to 99% accuracy in binary classifications (Alzheimer’s disease AD vs. Cognitively Normal and Mild Cognitive Impairment vs. Cognitive Normal) and 96% in multi-class classifications (AD vs. MCI vs. CN), highlighting their potential for strong and early AD diagnosis (Parihar and Swami, 2024; Song et al., 2021) By combining structural and functional imaging data, these advancements support former interventions and better patient outcomes (Shukla et al., 2023) Table 3 depicts the comparison of single modalities with fusion modalities.

**Table 3:** Comparison of single modality (MRI, PET) and fusion approach (MRI-PET Fusion) for the early diagnosis of Alzheimer’s Disease.

Modality	Study Findings	Evidence
MRI	MRI is effective in detecting brain atrophy and volumetric changes, specifically in the hippocampus and medial temporal lobe.	Rao et al. (2023)
	Demonstrates high sensitivity in detecting early structural changes in gray matter but limited specificity in differential diagnosis.	Chouliaras and O’Brien (2023)
PET	FDG-PET detects early metabolic changes in the posterior cingulate and precuneus regions, preceding structural atrophy.	Mosconi et al. (2004)
	PET using amyloid tracers (e.g., PiB) improves the detection of amyloid-beta plaques in preclinical stages of Alzheimer’s.	Suppiah et al. (2019)
MRI-PET Fusion	Combining PET and MRI enhances the detection of early biomarkers by integrating structural and metabolic changes.	Lee et al. (2024).
	Integration of PET and MRI data improves diagnostic accuracy through automated machine learning techniques.	Castellano et al. (2024)
	Demonstrates improved sensitivity and specificity in early detection by combining anatomical and functional data.	(Song et al., 2021)
	Fusion models using deep learning enhance early detection with improved performance compared to single modalities.	Venugopalan et al. (2021)
	Advanced neural networks combining MRI and PET achieve higher diagnostic accuracy in distinguishing Alzheimer’s from other dementias.	Huang et al. (2019)

The following Table 4 enlightens different multimodal fusion approaches combining MRI and PET imaging for the early diagnosis of AD.

**Table 4:** Multimodal fusion of MRI and PET imaging for detection of Alzheimer’s Disease (AD).

Fusion Technique	Description	Key Findings	Reference
Image-level Fusion	Combines MRI and PET images to create a single composite image. Example: “GM-PET” modality fuses gray matter tissue area of MRI with FDG-PET images.	Retains both structural and metabolic information; outperformed unimodal and feature fusion methods.	Song et al. (2021)
Feature-level Fusion	Extracts features from MRI and PET separately, then combines them. Example 1: Wavelet transform-based fusion with deep learning using ResNet-50.	Incorporates structural and metabolic data effectively for improved diagnosis.	Choudhury et al. (2024)
	Example 2: Pre-trained neural networks for 2D feature fusion of MRI and PET.	Enhanced feature extraction and fusion capabilities.	Suma et al. (2022)
Decision-level Fusion	Classifiers are trained on individual modalities (MRI, PET, EEG), and outputs are combined.	Ensemble classifier approach improved diagnostic accuracy by 10–20% compared to individual modalities.	Polikar et al. (2010)
3D Fusion Techniques	Operations include skull-stripping, image segmentation, and co-registration. Example: Comparison of 2D and 3D CNN architectures for MRI-PET fusion.	3D fusion achieved good accuracy (86.90%) for AD classification; 3D methods showed effectiveness.	Suma et al. (2022)

### 3.2 Functional connectivity and resting-state fMRI in AD diagnosis

Resting-state functional magnetic resonance imaging (rs-fMRI) has emerged as a promising tool for studying Alzheimer’s disease (AD) and its preclinical stages. Various studies have evidences that rs-fMRI can differentiate functional connectivity changes in the brain’s default mode network (DMN) across the spectrum of AD, from healthy aging to MCI and clinical AD (Binnewijzend et al., 2012; Damoiseaux, 2012). These variations are often characterized by diminished connectivity within the DMN, predominantly in the precuneus and posterior cingulate cortex, and are not dependent on cortical atrophy (Agosta et al., 2012; Binnewijzend et al., 2012) In the course of decreasing DMN connectivity in AD, some studies have detected enhanced connectivity in frontal networks, probably as a compensatory mechanism to preserve cognitive efficiency (Agosta et al., 2012).

Furthermore, AD patients have shown altered connectivity patterns between different brain areas, such as diminished positive correlations between prefrontal and parietal lobes and increased positive correlations within lobes (Ibrahim et al., 2021). These findings support the concept of an anterior–posterior disconnection phenomenon in AD. Thus rs-fMRI holds potential as a biomarker for monitoring of early detection and progression of disease. However, standardization of methodologies and analysis techniques is required for clinical implementation. Integrating rs-fMRI with other imaging modalities and cognitive measures may further improve diagnostic accuracy and provide deeper insights into AD pathophysiology (Damoiseaux, 2012).

### 3.3 Multimodal data preprocessing and alignment techniques

Multimodal data preprocessing and alignment techniques play a key role in the diagnosis of AD using neuroimaging data. Various studies have emphasized the importance of these modalities in refining diagnostic accuracy and elucidating meaningful features from different imaging modalities. Techniques like age correction, feature selection, and feature reduction enable meaningful analysis, with these methods enhance diagnostic accuracy by normalizing and reducing the dimensionality of data from various modalities, such as MRI, PET (positron emission tomography), CSF (cerebrospinal fluid) biomarkers, and genetic features (Lin et al., 2021). Some studies also focus on specific brain regions like the hippocampus for extracting discriminative features (Ahmed et al., 2019).

Though integration of multimodal data offers significant advantages, integrating high-dimensional imaging and genetic data poses challenges (Peng et al., 2019). To address these challenges, new approaches like structured sparsity and multiple kernel learning methods have been introduced to facilitate structured feature selection and fusion from mixed modalities. In conclusion, advanced preprocessing and alignment techniques, such as graph diffusion methods for enhancing similarity measures among subjects (Wang et al., 2023) and image fusion methods combining gray matter tissue areas of brain MRI and FDG-PET images (Song et al., 2021) have demonstrated improved performance in AD diagnosis. These advanced techniques improve data quality and integration, resulting in more precise and explainable diagnostic models for AD.

### 3.4 Challenges in integrating heterogeneous imaging data

The diverse range of imaging techniques, including CT, MRI, and PET, each offer unique understandings into AD pathophysiology but also poses complications in data integration (Johnson et al., 2012). These modalities provide varying types of information, from structural alterations to functional changes and molecular pathology, making it problematic to create a unified understanding of the disease process and progression. One significant challenge is the apparent paradox between the clinically diverse presentations of AD and the common underlying histopathological substrate.

One significant challenge is the apparent paradox between the clinically diverse presentations of AD and the common underlying histopathological substrate. Variant syndromes of AD, such as posterior cortical atrophy and logopedic variant primary progressive aphasia, exhibit different clinical phenotypes despite sharing similar pathological pictures (Warren et al., 2012). This heterogeneity complicates the interpretation of imaging data and highlights the need for a more refined understanding of how different brain networks are affected in AD (Loreto et al., 2024). To address these challenges, researchers are developing comprehensive knowledge bases like the Alzheimer’s Knowledge Base (AlzKB), which combines diverse data sources to provide a complete depiction of AD etiology and potential therapeutics (Romano et al., 2024).

Such approaches aim to capture the complex relationships between biological and pharmaceutical entities at various levels of organization. However, the successful integration of heterogeneous imaging data will require continued efforts to standardize data collection, develop advanced analytical techniques, and create frameworks that can accommodate the multifaceted nature of AD pathology and its clinical manifestations. Table 5 highlights the challenges in integration of heterogenous data.

**Table 5:** Challenges in integrating heterogeneous imaging data.

Challenges	Description
Diverse Insights from Multiple Imaging Modalities	CT (Computed Tomography): Detects structural abnormalities and excludes other causes of cognitive decline, such as tumors or significant brain atrophy. MRI (Magnetic Resonance Imaging): High-resolution imaging to detect hippocampal atrophy, a hallmark of AD. PET (Positron Emission Tomography): depicts molecular /functional data, e.g., glucose metabolism or amyloid and tau deposits. Each modality, i.e., CT, MRI and PET provides unique understandings but poses challenges for integration because of different spatial/temporal resolutions and biological targets (Zhang Y.-D. et al., 2020)
Challenges in Data Integration	Heterogeneity of data: Structural changes (e.g., atrophy) occur late, while functional/molecular changes manifest earlier but are harder to contextualize (Hofmann-Apitius et al., 2015) Variability in protocols and biomarkers: Differences in acquisition methods complicate the merging of diverse datasets (Nazir et al., 2024)
Clinical and Pathological Paradox	AD’s clinical diversity (e.g., posterior cortical atrophy, logopenic variant primary progressive aphasia) contrasts with its uniform pathology (amyloid plaques, tau tangles), creating challenges in linking imaging findings to varied clinical presentations (Jellinger, 2022)

In order to minimize the complexity of heterogenous data integration, additional researches are required to be directed for standardization of data collection methods across institutions and studies (Mueller et al., 2005). This will facilitate comparability and reproducibility of data. Furthermore, development of sophisticated analytical methods capable of combining data from multiple modalities (e.g., MRI-PET fusion) needs to be developed (Castellano et al., 2024). It is also required to create frameworks for data interpretation that can accommodate AD’s complexity and variability, incorporating clinical, imaging, genetic, and biomarker information (Li et al., 2015). Therefore, the integration of diverse imaging data in AD research holds the promise of providing a more holistic understanding of the disease. However, achieving this goal requires continued innovation in data standardization, analytical techniques, and interdisciplinary collaboration to address the multifaceted nature of AD pathology and its clinical presentations.

## 4 Predictive modeling of disease progression

Disease progression modelling is currently a key asset in most medical practices given that it assists clinicians and researchers to predict the course of a disease and respond adequately on preventive measures, diagnosis and treatment (Alowais et al., 2023). In particular, these models employ statistical and machine learning approaches to gather an enormous amount of data, describe its distribution, and forecast potential further events for specific patient or multiple patients at once (Raza and Abbas, 2024; Babb de Villiers et al., 2020). Of all the chronic and progressive diseases, they are highly essential for several diseases like cancer, cardiovascular diseases, diabetes and neurodegenerative diseases where timely intervention can greatly determine the state of the patients (Raza and Abbas, 2024; Wang J. et al., 2024).

It is a machine learning model, for instance, random forests, support vector machines (SVMs), and deep neural networks which deal with the data sourced from Electronic Medical Records, Imaging scans, Genomic data, wearable devices with a high degree of certainty (Raza et al., 2024b; Raza and Abbas, 2024; Du et al., 2020). For instance, in neurodegenerative diseases such as Alzheimer’s predictive models evaluates clinical and imaging biomarkers, longitudinal data on cognition and brain imaging to determine risk of further deterioration or progression from probable MCI to dementia (Du et al., 2024b). Temporal modelling is a type of predictive modelling that focuses on time series, disease trajectory (Bilgel et al., 2017).

Functions like RNNs and long short-term memory networks (LSTMs) are highly adept at identifying variations, and therefore are suitable for conditions that have over time varying biomarkers or symptoms (Othman et al., 2024). For instance, temporal models are used to forecast the growth rate of the tumor in cancer or blood sugar levels in diabetes to allow the clinician to prevent emergent complications on the basis of treatment (Sharma et al., 2017). Risk assessment is another of the most important applications of predictive modeling, which sorts patients by their risk level or the degree of risk that they experience adverse outcomes (Vadapalli et al., 2022). There is always some stratification to help identify patients for closer observation or immediate action. In addition, predictive models can accommodate patient-specific characteristics, including genetic attributes or lifestyle, and determine specific interventions possible for the patient with the disease (Tong et al., 2024).

Nonetheless, predictive modeling has some limitations such as data quality problems, a shift in dataset distribution, or high dimensionality, and requirements for sample size and variability (Lombardo et al., 2024). However, the interpretability of the learned model needs to be guaranteed for it to be useful in the clinical settings. Due to the growing complexity of these models, to make them more understandable and to build trust in such models, Explainable AI, or XAI, methodologies are being researched (Wyatt et al., 2024). Subsequently, as the field of predictive modelling progresses, the capability of incorporating online data from wearable devices and digital health applications will expand the usability of the model to become a more proactive field of healthcare.

### 4.1 Deep learning for predicting disease trajectory and biomarker identification

Therefore, among the most revolutionary instruments of early disease trajectory prediction and prognostic biomarker discovery is made possible by deep learning (DL), which falls within the field of artificial intelligence (Miller et al., 2024). AI will play a vital role in deciphering the mechanisms of disease, forecasting disease progression, and developing targeted therapies, thanks to its ability to analyse extensive and complex datasets rapidly and autonomously (Abbas et al., 2019; Zhang and Shen, 2012).

Deep learning extracts informative patterns from multimodal data such as medical imaging, genomics, and electronic health records (EHR) by leveraging more advanced architectures such as CNN, RNN, and Transformers (Li et al., 2020). Deep learning models use longitudinal data to analyze patterns and make predictions about future health trajectories (Du et al., 2020). As for neurodegenerative diseases such as Alzheimer’s, linear convolutional networks (LCNs) can manage brain imaging data to uncover early structural changes, while RNNs, for analysis of temporal data (for instance, cognitive scores and levels of biomarkers) can be used to predict disease progression by capturing the temporal dynamics (Alsubaie et al., 2024). They are also being applied to diabetes treatment, where deep learning models analyze continuous glucose monitoring data to predict changes in blood sugar levels, allowing for early medical interventions (Faruqui et al., 2019). Biomarkers have become increasingly essential to diagnostics and targeted treatments, and for the successful identification, deep learning has also emerged as a key parameter (Echle et al., 2021).

Deep learning models can derive potential biomarkers from genomic, proteomic, or metabolomic data that indicate the onset or progression of a disease event (Mann et al., 2021). For example, autoencoders and deep generative models may capture genomic variations present but often overlooked by DNA sequencing (e.g., somatic mutations in cancer) or less frequently examined patient specimens (e.g., rare syndromes), CNNs apply deep learning to histopathological images of solid tumors to examine cancer-specific markers (Guleria et al., 2023).

The fusion of multimodal data also demonstrates the deep learning ability across single/multiple factors (Li et al., 2022). Models trained on imaging plus genetics plus whatever clinical records can discover complex relationships and interactions, delivering a holistic view of disease dynamics (Kuznetsov et al., 2013). This is particularly useful for complex diseases such as cancer and autoimmune diseases, where altered pathways contribute to disease progression (Guleria et al., 2023). Medical deep learning brings the advantages but also challenges. This requirement of large labeled datasets can often be a bottleneck in training a model, and bias in the data can impact generalizability (Paproki et al., 2024).

Another issue is interpretability since most of deep learning models are “black boxes” and it is not easy to understand how the predictions are made (Paproki et al., 2024). To overcome these challenges there is required a joint effort of data scientist, clinicians, and regulatory bodies to ensure that models are indeed accurate, reliable, and ethical (Guan, 2019). The progress has been sustained in understanding the disease prognosis and biomarker discovery by deep learning enabled through computing capabilities and availability of data. When applied to health care, its uses promise early diagnoses, right treatment, and better results offering directions to precision medicine.

### 4.2 Temporal modeling for tracking cognitive decline

Temporal modeling is a particularly effective approach for tracking the specific cognitive changes that define AD, Parkinson’s disease, and other forms of dementia (Kunst et al., 2019). Temporal models come in handy in analyzing longitudinal data in that, they can identify change in cognitive function, biomarkers, or many other clinical assessments with time. They are also useful when it comes to diagnosing conditions at their early stage, as well as tracking the progression of a disease, and delivering personalized treatment (Zuidersma et al., 2019).

The modeling in this context involves adoption of various machine learning techniques especially the RNNs and its improved variants including, Long Short-Term Memory Networks (LSTMs) and Gated Recurrent Units (GRUs) (Monner and Reggia, 2012). Due to their suitability for processing sequential data, these models are rather useful for analyzing time series data, such as repeated strategic thinking test results, neuroimages, biomaterial values, etc. (Othman et al., 2024). For instance, LSTMs in modelling the decline in memory and executive function by incorporating across time-dependent data for better forecasts on future cognitive status (Zuidersma et al., 2019).

A major advantage of temporal modeling is that a large and diverse number of data sources can be incorporated: structural and functional neuroimaging, genomic and other molecular profiles, CSF biomarkers, and HER (Behrad and Saniee Abadeh, 2022). The holistic approach also allows for understanding cognitive changes and making prognosis concerning transitions from MCI to AD or other further stages of neurodegenerative disorder (Lombardo et al., 2024). For instance, integrating MRI with tau and amyloid beta biomarkers, makes it easier for the model to identify more sensitive changes in the brain’s structure and function (Behrad and Saniee Abadeh, 2022). Personalized temporal modelling extends the prediction mechanism by incorporating subject specific differences. Depression, age, type 2 diabetes, hypertension, smoking, obesity, APOE genotype, education, income, and occupation, are some of the predictors of progressive decline and capturing this data is necessary for accurate prognosis at the individual level (McFall et al., 2019).

Temporal constructors within the clustering techniques can also generate groups of patients sharing similar disease progress for better categorization of patient populations for clinical trials or for targeted treatment (Cui et al., 2023). The temporal modeling remains a problem in the following ways: dealing with gaps in available data, requiring vast amounts of data collected over time, and interpretability of prediction (Liu et al., 2023). Data imputation prevents data loss, while transfer learning applies existing algorithms to a new dataset, improving model effectiveness for clinical uses. This can be achieved while temporal modeling advances and becomes connected with other new constantly monitoring techniques such as wearables that can alert for Cognitive decline at even earlier stages than the current status (Arevalo-Rodriguez et al., 2021). They all have potential for helping decrease the load of the neurodegenerative ailments and enhance individual experiences of patients.

### 4.3 Using deep learning for MCI vs. AD stage differentiation

Deep learning has been shown to be a helpful method for distinguishing between AD and mild MCI, two critical stages in the continuum of cognitive decline and an area where the differential diagnosis of neurodegenerative diseases has been chronically understudied (Grueso and Viejo-Sobera, 2021). While MCI is a mild cognitive disability that makes people develop dementia over time, AD is a severe cognitive disability that entails severe memory impairment, disability, and general neuronal loss. It is very important to be able to differentiate between these stages correctly so that interventions may be addressed appropriately (López et al., 2020).

CNNs are considered best suited for deep learning models when addressing neuroimaging data including MRI and PET scans (Tufail et al., 2022). These models also exclude hierarchical features from imaging data that reflect the structural and functional brain changes related to MCI and AD. CNNs can spot early atrophy in areas like hippocampus and entorhinal cortex in which moderate AD development occurs (Woźniak et al., 2021). This automated feature extraction helps bypass the preprocessing step required for each scan and greatly upgrade diagnostic thoroughness (Jin and Shi, 1998). The temporal modeling part helps analyze eventual transitions from MCI to AD, which proves the dynamic nature of neurodegeneration (Bilgel et al., 2017). A more advanced differentiation is achieved by multimodal deep learning that combines different data feeder. Thus, adding neuroimaging with other structural and functional data, including genetic profiles (for example, APOE status), cerebrospinal fluid biomarkers (for instance, amyloid beta and tau), and clinical evaluation enhances the coverage of knowledge about the disease (Venugopalan et al., 2021). These multimodal models are typically better than single-modality ones because they combine different type of data.

However, there are challenges when employing deep learning for the differentiation between MCI and AD. A major challenge is the requirement for large annotated data sets to be able to ‘teach’ the models well (Li et al., 2015). Additionally, the “black box” nature of deep learning models can limit their clinical acceptance. In response to this, it is possible to design explainable artificial intelligence (XAI) methods that will enhance the understandability of the model’s prediction to the clinicians (Chaddad et al., 2023). There has also been recent progress in fine-tuning such models to work with small datasets in order to build models that work well with limited resources (Agarwal et al., 2021). With the help of deep learning, the study of MCI and AD can be differentiated at an earlier stage and the focus can be shifted to accurate early diagnosis, as well as identifying more individual approaches to prologue patients with neurodegenerative diseases (Raza and Abbas, 2024).

### 4.4 Risk stratification and personalized treatment approaches

Risk stratification and targeted management on the other hand have become part of precision medicine as it brings out who among the people has different level of risk for something and how treatment option should be administered to the patient (Reddy et al., 2020). It enables the identification of who among the population is at different extents of disease danger and how treatment should be implemented to the patient.

These treatments are particularly important in chronic diseases including cardiovascular diseases, cancer, diabetes and neurodegenerative diseases (Xu et al., 2021). Risk stratification means placing patients into categories that are expected to develop disease or other adverse outcomes along a specific continuum (Jin et al., 2020). It relies on data obtained from clinical practice, genomic and biomolecular, and patient’s individual parameters and behaviors to produce customer-specific risk models (Jin et al., 2020). This means “risk stratification” that may require blood levels of cholesterol, blood pressure, family history, and in some cases also genetic risks to calculate for example the probability of myocardial infarction or stroke as for cardiovascular disease (Paquette and Baass, 2018).

Machine learning models go further and analyze big data to identify nonlinear patterns in the data that improve the accuracy of risk predictions and allow for the early identification of high-risk individuals (Mariani et al., 2021). Personalized treatment strategies go beyond risk stratification by creating tailored interventions based on a person’s individual characteristics. In oncology, for instance, genetic profiling of tumors enables the use of targeted therapies aimed at certain mutations, including HER2 inhibitors for breast cancer patients with HER2 overexpression (Oh and Bang, 2019).

In psychiatry, for example, we can select antidepressants based on genetic variants that change drug metabolism, a process that should reduce side effects and improve effectiveness (Tansey et al., 2012). The strategies could be potentiated by the integration of wearable technologies and digital health platforms permitting continuous monitoring of physiological parameters towards real-time risk stratification and progressive updating of therapy (Lu et al., 2020). Continuous glucose monitoring systems, for example, offer real-time feedback that can inform personalized dials for insulin dosing for patients with diabetes, reducing the risk for complications such as hypoglycemia (Breton et al., 2018).

Despite the fact that the leverage of risk stratification, and tailored treatments open up large avenues, its business is framed by several major hurdles for ‘big data’-driven research, including data protection, inequality in access to advanced technologies, and the necessity of solid clinical backing for the adoption of predictive models and personal drugs (Parikh et al., 2019). Moreover, for machine learning algorithms to be widely adopted they must be interpretable and create confidence in their outputs (Rudin et al., 2021).

AI and genomics advances are about to take the burden off of personalized medicine delivery challenges (Zahra et al., 2024). Focusing on targeted care in such approaches transforms healthcare from a reactive paradigm that limits disease prevention, therapeutic improvements, and resource allocation, to an anticipative approach that envisages all of these parameters in a managed and systematic manner. So, risk stratification and personalized treatments will be the future of clinical practice, which should ultimately result in improved outcomes both at the patient and the population levels.

### 4.5 Deep radiomics in PET and MRI imaging for Alzheimer’s disease diagnosis

Deep radiomics is an AI-driven approach that in PET and MRI is revolutionizing AD diagnosis by extracting quantitative biomarkers that provide deeper insights into disease progression. By leveraging deep learning techniques, such as CNNs, radiomics can automatically extract high-dimensional features from imaging data, revealing subtle metabolic and structural abnormalities that may not be visually detectable (Stefano, 2024). The major advantages of deep radiomics in AD diagnosis include early detection of neurodegenerative changes, objective biomarker quantification, and the ability to assess disease progression longitudinally (Ding et al., 2021) Additionally, integrating radiomics with multimodal AI models that incorporate genetics, clinical data, and cognitive assessments can enhance diagnostic accuracy and patient stratification for clinical trials (Seo et al., 2022) However, challenges such as standardizing radiomics pipelines, ensuring data generalizability across diverse populations, and obtaining regulatory approval (e.g., FDA, CE Marking, GDPR compliance) must be addressed for widespread clinical adoption (Obuchowicz et al., 2025). Additionally, deep radiomics combined with explainable AI (xAI) techniques will refine AI-assisted diagnostic pipelines, making them more trustworthy, transparent, and clinically actionable for improving Alzheimer’s disease detection, prognosis, and patient care (Alharthi et al., 2024).

Tables 6 and 7 summarize the comparison of deep radiomics with traditional radiomics and benefits of deep radiomics in neuroimaging, respectively, for AD diagnosis. These AI-driven approaches improve the differentiation between normal aging, MCI, and AD, enhancing the ability to predict cognitive decline and personalize treatment strategies.

**Table 6:** Comparison of radiomics and deep learning in neuroimaging.

Aspect	Traditional radiomics	Deep radiomics
Feature Extraction	Predefined features (texture, intensity, shape)	Learned hierarchical features via CNNs (Zhou et al., 2021)
Data Interpretation	Limited to manual analysis	Integrates imaging with clinical/genetic/cognitive data (Shu et al., 2021)
Predictive Modeling	Relies on classical statistical models	AI-driven prediction of disease progression (Peng et al., 2025)

**Table 7:** Comprehensive overview of deep radiomics in neuroimaging for AD diagnosis.

Modality	Biomarker/Feature	Purpose/Application in AD	Deep Radiomics Benefit
FDG-PET	Glucose metabolism	Detects hypometabolism in AD-prone regions	Identifies subtle imaging patterns via automated feature extraction (Zhou et al., 2021)
Amyloid PET	Amyloid-beta	Identifies amyloid plaque accumulation	Enhances early diagnosis by detecting AD before clinical symptoms appear (Rasi et al., 2024)
Tau PET	Tau protein	Correlates with neurodegeneration and cognitive decline	Assists in predicting MCI to AD conversion (Feng et al., 2024)
Structural MRI (sMRI)	Brain atrophy	Measures hippocampal and medial temporal lobe atrophy	Detects minute volumetric changes using voxel-based analysis (Shih et al., 2024)
Functional MRI (fMRI)	Neuronal connectivity	Assesses activity and connectivity changes in AD	Identifies microstructural abnormalities via texture analysis (Leandrou, 2021)
Diffusion Tensor Imaging (DTI)	White matter integrity	Evaluates microstructural changes in neuronal pathways	Combines MRI with PET for enhanced diagnostic accuracy (Afrazeh, 2024)

### 4.6 Explainable AI (xAI) in Alzheimer’s disease diagnosis

With the increasing reliance on deep learning models for medical diagnostics, ensuring trust, transparency, and interpretability is crucial, especially in critical conditions like Alzheimer’s disease (Nazir et al., 2024). Traditional AI models, particularly convolutional neural networks (CNNs) and transformer-based models, often operate as black boxes, making it difficult for clinicians to understand, validate, and trust the decisions made by these models (Lai, 2024). xAI addresses this challenge by providing interpretability techniques that make AI decisions more transparent and reliable for clinical applications (Adeniran et al., 2024).

#### 4.6.1 Key xAI techniques for Alzheimer’s disease diagnosis

Several interpretability methods are applied to deep learning models used in Alzheimer’s disease detection from brain imaging (e.g., MRI, PET scans) and clinical data. Some of the most widely used techniques include:

1. Grad-CAM (Gradient-weighted Class Activation Mapping), generates heatmaps by identifying the most important regions in an image that influenced the model’s prediction (Song et al., 2024). It calculates the gradient of the predicted class with respect to the last convolutional layer and highlights relevant features in the input image (Asokan and Seshadri, 2023) this technique is used in CNN-based Alzheimer’s classifiers to highlight key regions in MRI or PET scans that contribute to the prediction (e.g., hippocampal atrophy). It also helps radiologists to validate AI predictions and ensures that the model focuses on medically relevant areas rather than noise (Toumaj et al., 2024).
2. SHAP (Shapley Additive Explanations): SHAP values are derived from game theory to assign an importance score to each feature in a model’s prediction. It explains how each input feature (e.g., age, brain volume, cognitive test scores) contributes to the final decision (Braithwaite et al., 2020). In AD these techniques are used in tabular models (e.g., decision trees, deep learning) to analyze biomarker data, clinical assessments, and genetic risk factors. It facilitates clinicians to understand which features (e.g., APOE4 genotype, CSF biomarkers) are most influential in predicting disease progression (Sarica et al., 2024).
3. LIME (Local Interpretable Model-agnostic Explanations) perturbs the input data and observes how predictions change, fitting a simpler, interpretable model (e.g., linear regression) to explain the prediction locally (Zafar and Khan, 2021). In AD, it is applied to black-box classifiers to assess why an AI system labeled a brain scan as AD, MCI (Mild Cognitive Impairment), or normal aging. It helps in validating model predictions by ensuring consistency with known clinical insights. In AD, it is applied to black-box classifiers to assess why an AI system labeled a brain scan as AD, MCI or normal aging (Hasan Saif et al., 2024).

Clinical usability is strengthened by leveraging xAI techniques. This is done by improving trust as physicians are more likely to adopt AI-driven diagnostic tools when they can see and verify why a model makes specific predictions. As far as Regulatory compliance is concerned, Explainable models align with healthcare AI regulations (e.g., FDA, GDPR in AI/ML) by making AI decisions auditable and interpretable. Additionally, xAI enhance patient outcomes by making personalized treatment possible as clinicians can understand AI predictions and make informed interventions based on transparent reasoning (Vimbi et al., 2024).

## 5 Challenges in data standardization and generalization in imaging

Variability in imaging protocols and data quality presents significant challenges in applying imaging technology across different fields, including medical imaging, remote sensing, and scientific imaging. Typically, radiological results are assessed qualitatively by the human eye and expert interpretation (Gao et al., 2019). However, quantitative assessment can provide more valuable insights that are often missed by subjective human evaluation. For personalized treatment, objective assessment becomes crucial for accurate quantification. A major issue is the variability in results, which complicates generalization. This variability arises from several factors (Zeng et al., 2023).

The main challenges of deep learning models in detecting early AD are their interpretability and limitations in clinical adoption due to lack of explainability (Vimbi et al., 2024). Another important thing is that, unlike traditional machine learning models that produce clear decision boundaries, deep neural networks act as complex “black boxes” making it hard to understand the logic behind their predictions (Vimbi et al., 2024). This lack of transparency unsettles clinicians and regulators, because the decision process is opaque, so it is not possible to validate whether models are finding biomarkers related to the disease, or whether they are being influenced by irrelevant correlations, such as scanner artifacts or demographic biases.

Recent advancements in explainable AI (XAI) techniques have been proposed to improve model interpretability to overcome these challenges (Machlev et al., 2022). Techniques like Gradient-weighted Class Activation Mapping (Grad-CAM), SHAP, and LIME provide visualizations of the most salient brain segments that inform model predictions, leading clinicians to better evaluate the reasonableness of AI outputs (S Band et al., 2023). The introduction of attention mechanisms in transformer-based models also show the potential of such an approach to enhance the interpretability of predictions mapping MRI and PET scans directly to disease-relevant biomarkers (Sibilano et al., 2024). Furthermore, hybrid models that combine deep learning with manually crafted radiomic features like hippocampal atrophy serve to unify AI-based findings and known clinical relevance, as both diagnostics can be used together to explain the outputs of deep learning-derived models (Adadi and Berrada, 2018; Xu et al., 2024). These approaches can alleviate resistance towards black-box models, by providing visual or quantitative explanations regarding decision-making processes.

In addition to explainability, uncertainty estimation techniques (e.g., Bayesian deep learning) can help quantify the confidence level for various AI predictions (Nor et al., 2022). This is especially useful for clinical applications, as healthcare professionals can balance AI-generated diagnoses in light of the model’s confidence level (Sai et al., 2024). In addition, interactive AI systems in which clinicians query and refine outputs from the model may be an intuitive way to increase trust and usability (Nasarian et al., 2024). Empirical investigations further support dataset curation and improvement with annotated lesion maps, further directing deep learning models to relevant, biological features and lessening potential dependencies on unrelated features or patterns (Rai et al., 2024). Importantly, benchmark drives within the research community for interpretability will be critical for real-world clinical deployment of future models with the levels of transparency required.

### 5.1 Diversity in imaging protocols and data quality

The diversity of imaging protocols and data quality can lead to inconsistencies that affect the accuracy, dependability, and precision of the results. Imaging protocols encompass the steps, methods, and settings used to acquire imaging data. Key factors contributing to variability in imaging protocols include (Keenan et al., 2021).

- Imaging Type: Different types of imaging techniques can be employed, such as MRI, CT scans, X-rays, ultrasounds, or satellite imagery (Hussain et al., 2022).
- Device Parameters: Variations in exposure time, resolution, contrast, and scanning parameters can impact the quality of the obtained images.
- Data Processing: The final steps in processing the captured data can influence the outcome.

The variation in imaging protocols is influenced by factors such as the type of equipment used, operator differences, institutional standards, and technological advancements. For instance, different organizations may use different models of MRI machines with distinct hardware, software, and scanning protocols. Even within the same institution, two machines might vary due to differences in magnetic field strength, coil types, and scan sequences. These inconsistencies make it difficult to standardize and compare images across devices (Hussain et al., 2022). Then comes the quantitative imaging biomarkers, these are quantitative figures from pictures that can describe the nature of physiological developments, pathologies or treatment comebacks (Radwan et al., 2024).

The major concepts related to imaging biomarkers are:

- Transition to digital imaging
- Standardization and control of imaging
- Accuracy and precision in results
- Reliability and reproducibility (generalizability)
- Statistical tools for assessing variability
- Variation in MRI and CT scam along with the sources of variation
- Computer aided software used in imaging (Wood, 2020).

#### 5.1.1 Human factors and technological advancements

Human factors contribute significantly to variability in imaging results. Differences in operator training, experience, and expertise can lead to divergent approaches to data acquisition and analysis. Additionally, while new technologies generally improve imaging quality, older technologies become outdated, further complicating standardization efforts. Variations in machine settings—such as resolution, scanning time, and slice thickness—also affect image quality (Hussain et al., 2022).

#### 5.1.2 Standardization efforts

To address these challenges, significant efforts have been made to standardize imaging protocols and results across institutions and devices. One such initiative is the Digital Imaging and Communications in Medicine (DICOM) standard, which aims to ensure high-quality imaging and protocol standardization (Hussain et al., 2022). Additionally, advancements in artificial intelligence (AI) and machine learning are being leveraged to automatically adjust imaging factors, optimizing image quality regardless of the equipment used.

### 5.2 Addressing small sample sizes and imbalanced data

Learning from imbalanced data remains a complex issue in machine learning and data mining. Despite two decades of research on this topic, the challenge persists, especially in binary classification tasks. The advent of big data has provided insights into imbalanced learning, but it has also introduced new challenges (Sandfort et al., 2019). Hybrid approaches—combining data-level and algorithm-level methods—are becoming more prominent in the field. Current approaches focus not only on the division of data but also on the inherent difficulties associated with the nature of the data itself (Krawczyk, 2016). The presence of small cohorts in machine learning is an important consideration which can reduce the reliability, statistical power, and generalization of the model and can cause overfitting that occurs when the model captures noise instead of the original patterns, leading to poor presentation to resolve this issue expansion of dataset is needed while including methods such as random forest to improve quality (Kokol et al., 2022). However, implementing nested cross-validation can reduce biases even with limited data. Moreover, a careful model selection can also prevent overfitting in small sample sizes (Vabalas et al., 2019).

### 5.3 Generalizability across diverse populations and settings

Inferential goals in research typically involve gaining insights into a particular target population based on study observations. However, the results can vary depending on the chosen population. In some cases, such as randomized controlled trials (RCTs) or policy research, the sample may not truly represent the target population (Zhang et al., 2020b). Clinicians, in particular, may face challenges in making prognostic evaluations and can be prone to inaccuracies in their conclusions (Korevaar et al., 2023). Reproducibility, defined as the ability to generate precise predictions across individuals not part of the study, remains a key challenge (Degtiar and Rose, 2023). Further, working on extensive and balanced datasets will reduce bias and increase the generalizability of findings across different cohorts and adapting self-directed models can help in increasing model performance, which can then detect diseases like chronic obstructive pulmonary disease (COPD) across different ethnic groups (D Almeida et al., 2024). The use of models that are specific to definite settings can enhance the outcomes and reliability, especially in locations where data is frequently available (Yang et al., 2023).

### 5.4 Handling missing data and noise in multimodal datasets

Multimodal learning, which combines insights from different data sources, is increasingly applied to address real-world challenges, such as noisy or incomplete data (Cao et al., 2022). Despite this, current research lacks a deep investigation into how noise and data incompleteness affect prognostic outcomes in multimodal datasets. Understanding the impact of these factors is essential for improving the accuracy and reliability of predictions derived from multimodal data sources (Degtiar and Rose, 2023).

#### 5.4.1 Challenges in deploying AI models for Alzheimer’s disease diagnosis

While AI-driven models show promise in diagnosing Alzheimer’s disease (AD), several hurdles must be addressed before they can be widely adopted in clinical settings. These include regulatory approval, real-world validation, and seamless integration into existing workflows. Table 8 summarizes that for AI to be effectively deployed in Alzheimer’s disease diagnosis, it must be regulatory-compliant, validated in real-world settings, and seamlessly integrated into clinical workflows. Overcoming these hurdles will ensure that AI serves as a trustworthy, efficient, and clinically useful tool in early detection and patient care.

**Table 8:** Key challenges and practical implications of AI models.

Hurdle	Challenges	Potential Solutions
Regulatory Approval	AI models must comply with strict healthcare regulations (e.g., FDA, CE Marking, GDPR in AI/ML) (Pantanowitz et al., 2024)	Develop explainable AI (xAI) models to enhance transparency and regulatory acceptance (Gaur and Gaur, 2024)
	Variability in data sources (MRI/PET scans, biomarkers) leads to model inconsistencies (Stefano, 2024)	Conduct standardized multi-center trials to validate model reliability (Kumar et al., 2024)
Real-World Validation	AI models trained on research datasets often fail in diverse patient populations (Daneshjou et al., 2021)	Perform external validation on real-world hospital data to assess generalizability (Birkenbihl et al., 2020)
	Lack of longitudinal studies hinders AI’s ability to track disease progression (Habibi et al., 2025)	Integrate AI with longitudinal datasets to improve early detection and monitoring (Kale et al., 2024)
Integration into Clinical Workflows	AI tools must seamlessly work with existing Electronic Health Records (EHRs) and PACS (Picture Archiving and Communication Systems) (Kaur and Sharma, 2024)	Develop interoperable AI solutions compatible with major healthcare IT systems (Adegoke et al., 2025)
	Physicians may lack trust in AI predictions due to the black-box nature of deep learning (Lai, 2024)	Incorporate explainable AI techniques (Grad-CAM, SHAP, LIME) to improve clinician confidence (Tuan, 2024)
	Time constraints in clinical practice limit AI adoption (Adler-Milstein et al., 2022)	Design AI systems with user-friendly interfaces that provide rapid, interpretable insights (Li et al., 2024)

### 5.5 Future directions (these lines needs to be added in existing conclusion)

To enhance Alzheimer’s AI models, future research should focus on, combining multiple xAI techniques for better interpretability, integrating multimodal data (imaging + genetics + clinical features) for a comprehensive diagnosis and developing interactive AI interfaces that allow doctors to query and adjust AI-driven predictions.

## 6 Conclusion

The integration of deep learning with multimodal brain imaging marks a significant step forward in the early diagnosis and treatment of Alzheimer’s disease. By leveraging AI’s power to analyze complex datasets, researchers can achieve more accurate, timely, and personalized approaches to AD diagnostics and treatment. However, several challenges remain, particularly regarding data standardization, model generalization, and clinical applicability. Future efforts should focus on improving the interpretability of AI models and overcoming technical and ethical barriers to facilitate their broader adoption in clinical settings.
